# Construction and Building Materials: Highlights from the 2025 Editor’s Choice Collection

**DOI:** 10.3390/ma19194259

**Published:** 2026-10-08

**Authors:** Marco Corradi

**Affiliations:** Department of Engineering and Technology, University of Huddersfield, Huddersfield HD1 3DH, UK; m.corradi@hud.ac.uk

## 1. Introduction

The construction sector is undergoing a significant transformation driven by the combined demands of climate change mitigation, resource efficiency, infrastructure resilience and the need to adapt to rapidly changing societal and technological requirements. Cement, concrete, asphalt, mortars, composites and other conventional materials remain indispensable to the built environment, yet their production and use are associated with substantial consumption of natural resources, energy demand and environmental impacts [1,2,3]. Consequently, current research is increasingly moving beyond the traditional objective of achieving high mechanical properties and is addressing the overall material performance across the entire life cycle [4,5]. This includes the incorporation of secondary and recycled resources, reduction in embodied carbon, improved durability, resilience to aggressive environments, optimisation of material microstructure and the development of predictive approaches capable of linking material characteristics with structural performance [6,7,8].

One of the most important developments in construction materials research is the transition from a linear to a circular material economy [9,10]. Large quantities of industrial, agricultural, construction and demolition waste are generated every year, while natural aggregates and conventional mineral resources are becoming rare and expensive. The conversion of these wastes into functional construction materials therefore represents both an environmental opportunity and a technical challenge. Recent research has demonstrated the potential of steel slag, fly ash, biomass ash, ceramic waste, recycled concrete, phosphogypsum and waste polymers as constituents of cementitious and composite materials, but more research is needed before these can be used in construction. Such approaches can reduce the demand for virgin raw materials while simultaneously diverting waste from landfill and, in some cases, improving material performance.

The incorporation of waste materials, however, cannot be considered solely in terms of replacement ratios [11,12]. Their chemical composition, particle morphology, porosity, reactivity and compatibility with the cementitious matrix can highly influence hydration, microstructure and long-term performance [13,14]. In addition, their use presents challenges related to material variability, contamination, compatibility and processing requirements, as this may affect mechanical strength, durability, workability and long-term performance [15,16,17]. Further challenges include limited standardisation, quality control, environmental risks, technical and economic viability, and the feasibility of consistent large-scale implementation [18].

The articles collected in this Special Issue illustrate this complexity. Several contributions demonstrate that waste-derived constituents can provide significant advantages, including improved sulphate resistance, freeze–thaw durability, reduced permeability and lower environmental impact. At the same time, they highlight that careful optimisation and characterisation are important if recycled materials are to provide reliable alternatives to conventional ones.

Another major direction is the development of materials specifically designed to fulfil specific structural requirements. Pervious concrete provides an interesting example [19,20]. Unlike conventional concrete, its interconnected pore structure is often designed to facilitate water infiltration, making it attractive for sustainable drainage and sponge-city applications. However, increasing porosity generally reduces mechanical strength and generates other significant challenges. The challenge is therefore to optimise the material rather than maximise a single mechanical or rheological property. Similar multi-objective and multi-disciplinary considerations arise in lightweight concrete (structural and non-structural applications, lightness for reducing seismic forces, thermo- and acoustic insulation aspects, etc.), asphalt mixtures for railway applications and recycled polymer–gypsum composites, where mechanical performance, durability, thermal behaviour, permeability and environmental impact must be considered together [21,22,23,24].

Durability is another aspect emerging from the contributions. Extending the service life of infrastructure is important and represents an effective strategy for reducing its environmental impact because premature deterioration leads to additional material consumption, repair activities and energy use [25,26]. Concrete and cementitious materials are exposed to a wide range of environmental actions, including freeze–thaw cycles, fatigue, sulphate attack, temperature variations and moisture fluctuations. The resulting deterioration is often faster than estimated and governed by complex interactions between transport mechanisms, chemical reactions and microstructural changes. Understanding these aspects requires approaches that combine conventional mechanical testing, chemical and microstructural analyses, full-scale testing and dynamic-response assessment with advanced characterisation and monitoring techniques.

The increasing integration of experimental investigation, numerical modelling and data-driven interpretation represents another important advancement. Finite-element modelling can only provide insight into mechanisms, and it should be applied with caution, while in situ testing, imaging and nondestructive monitoring techniques can reveal damage evolution at different scales. Digital Image Correlation, acoustic emission monitoring, X-ray diffraction, scanning electron microscopy, computed tomography and mercury intrusion porosimetry are increasingly being combined with conventional destructive lab and in situ testing. Such multi-approaches provide a more complete understanding of how construction materials initiate damage, evolve under loading and ultimately fail [27,28,29].

The development of sustainable binders is an interesting research direction given the environmental footprint associated with Portland cement production. Supplementary cementitious materials and alkali-activated materials offer opportunities to reduce clinker consumption while exploiting industrial and agricultural residues. However, sustainability must be evaluated together with durability and long-term performance. The contributions addressing carbonated alkaline wastes, calcium-rich fly ash and wood-ash-based alkali-activated materials demonstrate how waste valorisation can be combined with targeted improvements in cementitious performance. These studies show that the structural behaviour of alternative binders does not depend only on their chemical composition, but also on curing, precursor chemistry, pore structure and exposure conditions.

Asphalt and bituminous materials are similarly undergoing significant development [30,31]. The increasing use of reclaimed asphalt pavement is interesting, as it provides a possible solution towards circularity in road infrastructure, but the performance of highly recycled mixtures depends on interactions between aged binder, virgin bitumen, rejuvenators and production conditions. Moreover, the application of asphalt materials to railway infrastructure introduces loading and environmental conditions that differ from those encountered in conventional road pavements. Optimising binder grade, modifiers, aggregate size and interlayer conditions is of great interest to ensure adequate stiffness, fatigue resistance, rutting resistance and durability.

The use of advanced composite materials also continues to expand in construction and infrastructure [32,33]. Lightweight sandwich structures, for example, combine high stiffness-to-weight ratios with efficient material use, making them attractive for transportation and other applications where weight reduction is critical. Nevertheless, their failure mechanisms can be highly localised. Accurate experimental observation and numerical modelling of such phenomena are therefore important for reliable structural design.

The twelve articles presented in this collection collectively demonstrate that advancement in construction materials is no longer driven by a single parameter. Instead, modern materials research is increasingly concerned with achieving an appropriate balance between structural performance, durability, resource efficiency, environmental impact and manufacturability. The studies also illustrate the importance of considering materials within their intended service environment rather than evaluating properties in isolation.

Taken together, this research provides a representative picture of current research aimed at creating construction materials that are more sustainable, resilient and technologically advanced. From recycled aggregates and waste-derived binders to modified asphalt, lightweight composites and advanced monitoring techniques, they exhibit innovation across construction materials research. Future progress will require continued integration of material science, structural engineering, numerical modelling, experimental characterisation and life-cycle assessment. Equally important will be the translation of laboratory findings into robust design methodologies, standards and construction practices. The articles in this collection contribute to this objective by addressing both fundamental material mechanisms and practical engineering applications, supporting the broader transition towards a more circular, durable and resource-efficient built environment.

## 2. An Overview of the Selected Articles

### 2.1. Pervious Concrete with Steel Slag Aggregate

The contribution by Liu et al. [34] addresses an interesting challenge in the design of pervious concrete: achieving sufficient hydraulic conductivity without compromising mechanical performance. The study is relevant to the development of sustainable drainage systems and sponge-city infrastructure. An interesting aspect of the work is the simultaneous consideration of target porosity, water-to-cement ratio and steel slag aggregate (SSA) replacement, providing an assessment of their respective influences.

The experimental results show that porosity is the dominant parameter governing the balance between strength and permeability. At the optimum water-to-cement ratio of 0.30, increasing porosity from 15 to 25% resulted in a substantial increase in permeability, but with a corresponding reduction in compressive strength. Also interesting is the finding that complete replacement of natural aggregate by steel slag can enhance strength, with the improvement reaching 37.86% at the lowest porosity.

This study demonstrates how industrial by-products can be incorporated into multifunctional concrete while addressing the competing requirements of structural and hydraulic performance. It provides a useful basis for the rational design of pervious concrete mixtures incorporating steel slag and contributes to the broader objective of converting industrial residues into value-added construction materials.

This research provides a novel integrated experimental and numerical assessment of how target porosity and water-to-cement ratio govern the strength–permeability relationship in pervious concrete and links porosity-dependent performance to distinct concrete failure mechanisms. The main strength is that it establishes validated empirical relationships and links porosity-dependent performance to distinct concrete failure mechanisms.

### 2.2. Early-Age Curing, Shrinkage and Microcracking

Bacharz et al. [35] investigate an important but sometimes underestimated aspect of concrete performance: the relationship between early-age curing conditions, shrinkage and the development of internal damage. Since shrinkage-induced cracking can significantly affect durability and serviceability, understanding the influence of environmental conditions is often important for reliable prediction of concrete behaviour.

The experimental programme considers C30/37-strength-grade concrete under different combinations of water curing, air drying and temperature variation. Shrinkage was monitored over 46 days, while acoustic emission (AE) measurements provided continuous information regarding damage development. An important finding is that the EN 1992-1-1 formulations provide a reasonable prediction of shrinkage for water-cured concrete but show discrepancies under air-drying and variable environmental conditions. This highlights a limitation of predictive formulas calibrated under controlled conditions when they are applied to the considerably more complex environments encountered in practice.

The relationship between shrinkage increments and acoustic-emission activity is also interesting. It suggests that AE monitoring could provide a nondestructive means of assessing early internal deterioration and potentially identifying conditions leading to cracking before visible damage develops.

The novelty of this study lies in correlating early-age concrete shrinkage with internal damage evolution using AE monitoring under non-standard temperature and humidity conditions. Unlike conventional frost-resistance studies, the research focuses on young concrete and demonstrates relationships between shrinkage strain, AE activity and microcracking, providing a potential approach to assessing early-age durability under adverse curing conditions.

### 2.3. Carbonated Alkaline Waste Materials in Eco-Blended Mortars

Moreno de los Reyes et al. [36] explore an innovative approach to reducing the environmental impact of cement-based materials by combining waste valorisation with accelerated carbonation. The study considers white ladle furnace slag, biomass ash and fine construction and demolition waste as partial replacements for Portland cement following carbonation treatment. This approach is interesting because it potentially addresses two environmental challenges simultaneously: the management of alkaline waste and the reduction of carbon emissions.

The research evaluates replacement levels of 7 and 20% and examines water absorption, electrical resistivity, compressive strength and microstructural characteristics. The results indicate that carbonated residues can be successfully incorporated into cement mortars without significant deterioration of their mechanical performance. Furthermore, reduced total and capillary water absorption was observed, indicating potential benefits for durability. Among the materials investigated, carbonated construction and demolition waste demonstrated the most favourable overall performance. The study consequently provides evidence supporting the use of carbonated alkaline residues as supplementary cementitious materials and contributes to the emerging concept of carbonation as both a waste-treatment process and a pathway towards carbon management in construction materials.

The innovative aspect of this study is the combination of carbonated alkaline waste materials with advanced multi-scale pore characterisation to develop lower-impact eco-mortars. By integrating mercury intrusion porosimetry and X-ray computed tomography, the study shows how carbonation modifies pore connectivity and structure, while linking these changes to hydration, water absorption, electrical resistivity and mechanical performance.

### 2.4. Asphalt for Railway Applications

Na et al. [37] address the application of asphalt materials in railway infrastructure. Although asphalt mixtures have been extensively investigated for road pavements, railway applications introduce different loading characteristics and performance requirements. The study therefore provides a useful assessment of binder and mixture characteristics relevant to asphalt concrete layers subjected to repeated railway loading.

The investigation demonstrates the importance of binder performance grade and modification. The study also examines crumb rubber modifier and styrenic thermoplastic elastomer modifications, with both providing substantial improvements in moisture and freeze–thaw durability.

The study is also interesting because it illustrates how established asphalt technologies can be adapted to infrastructure beyond conventional highways. The assessment of binder properties, mixture characteristics and testing methodologies provides useful information for the development of performance-based specifications for railway applications. Overall, the work highlights the potential of modified asphalt materials for durable railway infrastructure while demonstrating the importance of selecting binder grade, modification strategy and aggregate characteristics according to the specific loading and environmental conditions of service.

The innovation of this study lies in the evaluation of modified asphalt binders and mixtures, combining binder characterisation, mechanical performance, moisture resistance, freeze–thaw durability and wheel-tracking tests; it demonstrates the potential of polymer-modified binders, particularly styrenic thermoplastic elastomers and crumb rubber modifier, in improving rutting and moisture resistance in railway asphalt layers.

### 2.5. Reclaimed Asphalt Pavement and Production Conditions

Prosperi et al. [38] investigate the issue of the increasing use of reclaimed asphalt pavement (RAP): the influence of production conditions and virgin binder characteristics on the performance of highly recycled mixtures. The study is timely because increasing RAP contents provide an important pathway towards circularity in pavement construction, while potentially introducing greater variability in material behaviour.

The experimental programme considers mixtures containing 50% RAP and evaluates two virgin bitumen types, two rejuvenators, two production temperatures and three conditioning periods. Mechanical performance was assessed through stiffness, indirect tensile strength and cracking tolerance, while Fourier-transform infrared spectroscopy was used to investigate chemical changes in the binder.

The experimental results show that the performance of recycled mixtures cannot be determined solely by RAP content. The type of the virgin binder and the thermal history experienced during production play important roles in the resulting mechanical and chemical properties. Excessive temperature and prolonged exposure can promote undesirable ageing and potentially compromise the benefits associated with recycling.

The findings reinforce the principle that sustainable material solutions must retain adequate engineering performance throughout their service life. Optimising binder selection, rejuvenation and production conditions can therefore enable higher RAP contents while maintaining the mechanical and durability characteristics required for long-lasting pavement infrastructure.

The advancement in knowledge lies in quantifying the combined effects of virgin bitumen origin, hot mix asphalt production temperature and high-temperature conditioning time on RAP mixture performance. This study demonstrates how these often-overlooked variables influence binder ageing and rejuvenator effectiveness, while introducing a Temperature Index as a practical assessment tool.

### 2.6. Interlayer Shear Behaviour of Asphalt Pavements

Wang et al. [39] focus on the interlayer shear behaviour of asphalt pavements incorporating granular base layers, addressing the mechanism governing pavement deformation and reflective cracking. The study combines macroscopic experimental testing with a mesoscale finite-element model to represent the interaction between the granular base and asphalt surface course. The influence of binder type and content, asphalt mixture characteristics and external loading is systematically evaluated.

The results demonstrate the benefits of SBS-modified asphalt, which provides the highest interlayer shear strength, followed by SBR-modified emulsified asphalt and unmodified base bitumen. An optimum coating rate of 0.9 L/m^2^ is identified for SBS-modified asphalt. The analysis also shows that interlayer shear strength increases with applied load but decreases with increasing void ratio and nominal maximum aggregate size within the investigated ranges.

An interesting contribution of the work is the development of an equivalent macro–meso-predictive relationship. By linking microscale and mesoscale interaction mechanisms with engineering-scale performance, the proposed approach provides a potentially useful framework for pavement design and optimisation.

It should highlight the innovative approach of developing a macro–mesoscale framework that integrates finite element modelling with experimental shear testing to characterise asphalt pavement interlayer performance. This allowed to quantify aggregate interlocking and to incorporate important practical parameters—including bonding material application rate, air voids, aggregate size and external loading—into a validated predictive model for pavement design.

### 2.7. Durability Assessment of Cement Mortars with Recycled Ceramic Powders

Tokareva and Waldmann [40] study the potential of recycled ceramic powders to improve the durability of cementitious materials. While ceramic waste has long been recognised as a potential pozzolanic material, comparatively less attention has been devoted to its influence on long-term performance under aggressive environmental actions. The study addresses this gap by considering freeze–thaw exposure, elevated temperatures and sulphuric acid attack.

Test results show substantial improvements in freeze–thaw resistance for ceramic-containing mortars, with strength losses considerably lower than those observed in the reference mixture. The benefits also extend to elevated-temperature exposure. At 200 °C, some formulations incorporating ceramic powder exhibited strength gains, while ceramic-containing mortars generally showed improved resistance at 500 °C. Acid resistance provides another interesting aspect. The strength loss under 1% sulphuric acid was substantially reduced in the mortar containing ceramic powder, demonstrating the potential for ceramic waste to contribute to improved chemical durability. This research shows that waste-derived materials can provide not only a means of reducing cement consumption. Appropriate ceramic powders can modify the microstructure and improve resistance to several aggressive environments.

The interesting novelty of this study is the evaluation of ceramic demolition waste fineness, limestone filler and packing density in mortar durability across multiple aggressive environments. By combining these environments with multivariate statistical analysis, the research is able to define ceramic powder fineness as a fundamental parameter governing long-term durability.

### 2.8. Wrinkling of Sandwich Beams

Gashe et al. [41] investigate wrinkling in lightweight sandwich structures, a localised instability phenomenon. Sandwich composites are interesting because of their high stiffness-to-weight ratio, but their efficient structural performance depends on controlling local failure mechanisms in the thin skins and lightweight core. The study employs glass fibre–epoxy skins combined with a polymethacrylimide foam core and uses Digital Image Correlation (DIC), displacement measurements and force monitoring to investigate the onset of wrinkling under three-point bending. The experimental results are compared with analytical predictions and finite-element simulations. The numerical and experimental results show good agreement for compression wrinkling. However, the numerical model significantly overestimates the critical load for shear wrinkling. This discrepancy is particularly informative because it identifies limitations in conventional modelling approaches and highlights the complexity of shear-driven instability. More accurate representation of local imperfections, material behaviour and skin–core interaction may be required to improve prediction of shear wrinkling and enable more reliable design of advanced sandwich structures.

New knowledge lies in the experimental investigation and numerical modelling of shear wrinkling, a relatively underexplored failure mode in sandwich composite structures. The proposed testing methodology is able to adequately reproduce shear and compression wrinkling, while the numerical study shows the significant influence of mesh size on predicted wrinkling loads and wavelengths, highlighting limitations of conventional modelling approaches.

### 2.9. Fired Pumice Aggregates in Sustainable Concrete

Hokazono et al. [42] investigate the use of pumice as an alternative lightweight aggregate, addressing both the shortage of conventional aggregates and the management of large pumice deposits in southern Kyushu, Japan. The approach provides an interesting example of converting a potentially hazardous natural deposit into a useful construction resource. A new method is the firing treatment of pumice at 1000 and 1100 °C. The resulting aggregates demonstrate markedly different performance in concrete. Mixtures incorporating pumice fired at 1100 °C achieved compressive strengths up to 54.6 MPa, whereas the 1000 °C material resulted in lower strengths, even when cement content was increased. The findings indicate that the thermal treatment substantially influences the structural characteristics and mechanical performance of the aggregate.

The research illustrates the broader principle that sustainable aggregate development requires optimisation of processing and material selection. Locally sourced materials that may initially be unsuitable for structural applications can potentially become valuable construction resources following appropriate treatment. The contribution therefore provides an example of how regional material challenges can be transformed into opportunities for sustainable construction, reducing dependence on conventional aggregates while developing lightweight concrete with good mechanical and durability characteristics.

The originality of using fired volcanic pumice as a sustainable aggregate, and of linking firing temperature and particle-size optimisation to concrete strength and freeze–thaw resistance, is the main strength of this research. The results show that firing at 1100 °C can substantially enhance aggregate performance, enabling pumice concrete to achieve general-purpose strength while providing a potential solution to local landslide waste and global sand depletion.

### 2.10. Wood-Ash-Based Alkali-Activated Materials

Du et al. [43] examine the sulphate durability of wood-ash-based alkali-activated materials (AAMs), focusing on ensuring adequate long-term performance of alternative low-carbon binders. Alkali-activated materials have the potential to reduce reliance on Portland cement, but their durability under aggressive exposure conditions must be established before widespread structural application.

The study combines mechanical testing with X-ray diffraction and scanning electron microscopy to investigate changes occurring during sodium sulphate exposure. The results demonstrate that sulphate attack can cause decalcification and dealumination of hydration products, followed by the formation of expansive ettringite and gypsum. These processes result in microstructural deterioration, volume expansion and substantial strength loss.

The study therefore shows the complexity of designing waste-derived AAMs for aggressive environments. The chemical composition of the precursor system must be carefully balanced to control both initial performance and degradation mechanisms. By combining waste valorisation with improved durability, the research contributes to the development of more resilient low-carbon binders. The main advancement in knowledge can be identified in the evaluation of sulphate resistance of waste-ash-based alkali-activated mortars incorporating different binary precursors under severe cyclic sulphate exposure, by linking precursor chemistry and hydrate evolution to strength retention and microstructural durability, and by classifying distinct mechanisms by which aluminosilicate- and calcium-rich additions improve resistance.

### 2.11. Calcium-Rich Fly Ash and Sulphate Resistance

Aiming to reduce Portland cement consumption, Chousidis and Batis [44] investigated calcium-rich fly ash from the Ptolemais region of Greece as a supplementary cementitious material, considering sulphate resistance and long-term durability.

The results identify the relationship between early- and later-age performance. At early ages, fly ash incorporation reduces compressive, tensile and bond strengths because of its relatively slow pozzolanic reactivity. With extended curing, however, secondary reactions consume portlandite and generate additional calcium silicate hydrate, resulting in pore refinement and reduced permeability. This microstructural evolution is accompanied by improved mechanical performance and durability at later ages. Particularly noteworthy is the reported reduction in reinforcement corrosion rates. The bond tests also indicate a transition towards a more ductile response, suggesting potential benefits for repair materials where controlled deformation and adequate bond behaviour are important. The findings provide evidence that locally available calcium-rich fly ash can contribute to durable cementitious composites while reducing dependence on conventional Portland cement, particularly where long-term performance is prioritised.

The study advances current understanding by demonstrating that low-level (10%) calcium-rich Ptolemais fly ash can improve long-term cementitious performance despite early-age strength and bond reductions. It also highlights the combined influence of pore refinement, CaO-rich phase formation and permeability on carbonation and corrosion resistance, challenging explanations based solely on Ca(OH)_2_ availability.

### 2.12. Recycled Polymer–Gypsum Composites

Ferrández et al. [45] address the issue of plastic waste through the development of gypsum-based composites incorporating recycled polypropylene and high-density polyethylene pellets derived from discarded fishing nets. The study is relevant to the circular economy as it combines waste polymer recovery with the production of construction products suitable for prefabrication.

Recycled pellets were incorporated at replacement levels of up to 30% by volume while maintaining a constant water-to-gypsum ratio. An interesting conclusion is that, even at the highest replacement level, the composites achieved high flexural and compressive strengths.

The research therefore represents an interesting example of circular material design, in which a waste stream is transformed into a functional construction product. Future development should consider long-term durability, fire behaviour, recycling at end of life and large-scale manufacturing, but the results provide a promising foundation for the use of recycled polymer–gypsum composites in prefabricated construction.

An innovative contribution of this study is the development of gypsum composites incorporating up to 30% recycled polypropylene and high-density polyethylene pellets from fishing nets. The composites maintain sufficient mechanical performance while improving water resistance, reducing density and thermal conductivity, and lowering several environmental impacts, demonstrating their potential for circular-economy prefabricated building applications.

## 3. Conclusions

The twelve articles presented in this collection exhibit the evolution of construction and building materials towards solutions that combine performance, durability, resource efficiency and environmental responsibility. These studies highlight the potential of using industrial, agricultural, construction and polymer wastes as alternative constituents in concrete, mortars, alkali-activated materials, asphalt and gypsum-based composites. At the same time, they show that sustainable material development should be accompanied by the assessment of mechanical behaviour, durability and long-term performance. Advanced experimental techniques, including acoustic emission, Digital Image Correlation, computed tomography and microstructural analysis, together with numerical modelling, provide tools for understanding material behaviour across multiple scales. Several studies also demonstrate that optimisation of processing, curing, mixture proportions and exposure conditions is important for achieving reliable performance.

Across these 12 studies, a common characteristic is the development and validation of sustainable innovative construction materials and infrastructure solutions through waste valorisation, material optimisation and experimental approaches. Interesting advances include optimising steel-slag pervious concrete, linking early-age shrinkage with microcracking and using carbonated wastes in eco-mortars. Several of these studies also addressed recycled asphalt production, interlayer behaviour, ceramic-powder durability, sandwich-beam wrinkling and fired pumice aggregates. This Special Issue also presents interesting and innovative research on low-carbon binders, such as sulphate-resistant wood-ash AAMs and calcium-rich fly ash, and recycled fishing-net polymers for multifunctional gypsum composites.

These studies clearly contribute to circular, low-carbon and resilient construction materials, while highlighting the need for further experimental research to translate promising laboratory-scale developments into industrial engineering applications, standards and practice.
